# Cystatin C Shifts APP Processing from Amyloid-β Production towards Non-Amyloidgenic Pathway in Brain Endothelial Cells

**DOI:** 10.1371/journal.pone.0161093

**Published:** 2016-08-17

**Authors:** Xia-Fei Wang, Dong-Xin Liu, Yue Liang, Li-Li Xing, Wen-Hui Zhao, Xiao-Xue Qin, De-Shu Shang, Bo Li, Wen-Gang Fang, Liu Cao, Wei-Dong Zhao, Yu-Hua Chen

**Affiliations:** Department of Developmental Cell Biology, Key Laboratory of Cell Biology, Ministry of Public Health, and Key Laboratory of Medical Cell Biology, Ministry of Education, China Medical University, 77 Puhe Road, Shenbei New District, Shenyang 110122, China; Torrey Pines Institute for Molecular Studies, UNITED STATES

## Abstract

Amyloid-β (Aβ), the major component of neuritic plaques in Alzheimer’s disease (AD), is derived from sequential proteolytic cleavage of amyloid protein precursor (APP) by secretases. In this study, we found that cystatin C (CysC), a natural cysteine protease inhibitor, is able to reduce Aβ40 secretion in human brain microvascular endothelial cells (HBMEC). The CysC-induced Aβ40 reduction was caused by degradation of β-secretase BACE1 through the ubiquitin/proteasome pathway. In contrast, we found that CysC promoted secretion of soluble APPα indicating the activated non-amyloidogenic processing of APP in HBMEC. Further results revealed that α-secretase ADAM10, which was transcriptionally upregulated in response to CysC, was required for the CysC-induced sAPPα secretion. Knockdown of SIRT1 abolished CysC-triggered ADAM10 upregulation and sAPPα production. Taken together, our results demonstrated that exogenously applied CysC can direct amyloidogenic APP processing to non-amyloidgenic pathway in brain endothelial cells, mediated by proteasomal degradation of BACE1 and SIRT1-mediated ADAM10 upregulation. Our study unveils previously unrecognized protective role of CysC in APP processing.

## Introduction

Alzheimer's disease (AD) is the most common neurodegenerative disorder among the elderly population. Progressive accumulation of amyloid-β peptide (Aβ) in the brain parenchyma, caused by imbalance between Aβ production and clearance, is the primary mechanism driving AD pathogenesis [1]. In more than 80% of AD individuals, Aβ is deposited within cerebral vessel wall, termed as cerebral amyloid angiopathy (CAA) [2,3]. CAA was previously interpreted as the result of insufficient clearance of neuronal Aβ from brain parenchyma in AD because cerebrovascular system is the major pathway mediating brain Aβ elimination [4,5]. Recently, several groups reported endogenous Aβ generation in brain microvascular endothelial cells [6,7], suggesting an alternative endothelial-dependent pathway in Aβ deposition in CAA.

Aβ is generated from amyloid protein precursor (APP) through sequential proteolytic cleavage. There are two mutually exclusive pathways of APP processing, amyloidogenic and non-amyloidogenic pathway [8,9]. In Aβ-forming amyloidogenic route, APP is cleaved at its N-terminus by β-secretase to generate a membrane-bound soluble C-terminal fragment, and subsequent cleavage of this C-terminal fragment by γ-secretase produces Aβ peptides predominantly including Aβ40 and Aβ42 [8,9]. In the non-amyloidogenic pathway, APP is cleaved within the Aβ peptide sequence by α-secretase, producing a soluble N-terminal fragment named as soluble amyloid protein precursor α (sAPPα) [8,9]. The balance between amyloidogenic and non-amyloidogenic APP processing is critical to pathogenesis of AD. Proteolysis through the amyloidogenic pathway is associated with accumulation of the neurotoxic Aβ peptide [10], while the non-amyloidogenic pathway not only prevents the Aβ production, but also generates sAPPα that exhibits neuroprotective properties [11,12].

Cystatin C (CysC), also known as γ-trace, is a 13-kDa secreted cysteine protease inhibitor ubiquitously expressed in all nucleated cells and presented in all body fluids [13]. CysC plays various roles in many pathological processes, including tumor metastasis, atherosclerosis, inflammatory responses and immunomodulation [13]. CysC is highly abundant in brain tissue and the alteration of CysC levels in the cerebrospinal fluid (CSF) of neurodegenerative diseases have been reported. Recently, the protective role of CysC in Aβ deposition in AD is emerging [14]. In clinically diagnosed AD patients, the levels of CysC in the CSF are reduced compared to the non-dementia controls [15]. CysC could interact with Aβ [16,17] and this interaction results in a concentration-dependent inhibition of Aβ fibril formation [17] as well as inhibition of Aβ oligomerization [18,19].

Interestingly, a novel role of CysC in intracellular APP processing was revealed in this study. We found CysC is able to shift the amyloidogenic APP processing to non-amyloidogenic pathway, causing reduced Aβ40 and increased sAPPα secretion in brain endothelial cells. Furthermore, the inhibition of Aβ40 production is mediated by CysC-induced degradation of β-secretase BACE1 (β-site APP cleaving enzyme 1) through ubiquitin/proteasome pathway. The increased sAPPα secretion is caused by upregulation of α-secretase ADAM10 (a disintegrin and metalloproteinase 10) by CysC via SIRT1 (silent information regulator 1) in brain endothelial cells.

## Materials and Methods

### Cell Culture

The human brain microvascular endothelial cells (HBMEC) was provided by Dr. Kwang Sik Kim (Johns Hopkins University School of Medicine). HBMEC were cultured in a humidified atmosphere of 5% CO_2_, 95% air at 37°C, in RPMI 1640 medium supplemented with 10% fetal bovine serum (Invitrogen, Grand Island, NY), 10% Nu-serum (BD Biosciences, Franklin Lakes, NJ), 2 mM glutamine, 1 mM sodium pyruvate, 1×non-essential amino acids and 1×MEM vitamin. HBMEC were pre-incubated with CysC (Calbiochem, Darmstadt, Germany) 1 hr before addition of hydrogen peroxide (H_2_O_2_, Alfa Aesar, Lancs, UK). For experiments with inhibitors, HBMEC were pre-incubated with MG132, chloroquine or NH_4_Cl (Beyotime, Shanghai, China) before CysC and H_2_O_2_ treatment.

### Western Blot Analysis

HBMEC (2×10^6^/dish) were washed twice with ice-cold PBS and lysed with RIPA buffer (50 mM Tris–HCl, 150 mM NaCl, 1% NP-40, 0.5% deoxycholate, 0.1% sodium dodecyl sulfate) containing protease inhibitor cocktail (Roche, Mannheim, Germany). Cells were harvested by scraping and lysed on ice for 30 min. The lysates were centrifuged for 15 min at 12000×g at 4°C. The supernatant was collected and protein concentration of each sample was quantified using BCA protein assay kit (Thermo Scientific, CergyPontoise, France). Equal amounts of samples were separated by SDS-PAGE and transferred to PVDF membranes (Millipore, Billerica, MA). The PVDF membranes were blocked with 5% nonfat milk and incubated with the primary antibody at 4°C overnight. Then the blots were incubated with a horseradish peroxidase (HRP)-conjugated secondary antibody (Santa Cruz Biotech, Santa Cruz, CA) for 1 h at room temperature. Immunoreactive bands were visualized by Super Signal West Pico Chemiluminescent Substrate (Pierce, Rockford, IL) using LAS-3000mini imaging system (Fuji Film, Tokyo, Japan). The antibodies recognizing BACE1, BACE2, NICASTRIN, PS1, PS2, APH-1, PEN-2 and ADAM10 were obtained from Abcam (Cambridge, MA). Anti-SIRT1 was from Millipore and anti-ubiquitin was from Cell Signaling Technology (Danvers, MA). For quantitative analysis, the mean density of each band was measured by Image J software, and the band density of the interested protein was divided by the density of the corresponding loading control band to obtain the normalized values. Data are plotted as percentages of the control.

### Enzyme-Linked Immunosorbent Assay (ELISA)

The concentrations of Aβ40 and sAPPα in the culture medium of HBMEC were determined with ELISA kits (IBL, Gunma, Japan) according to the manufacturer’s instructions.

### Real-Time Reverse Transcription (RT)-PCR

The total RNA isolated with TRIzol reagent (Sigma-Aldrich, St. Louis, MO) was reverse transcribed using Moloney murine leukemia virus (M-MLV) reverse transcriptase (Promega, Madison, WI). Real-time PCR was performed on an ABI 7500 real-time PCR system (Applied BioSystems) with a SYBR premix Ex Taq kit (Takara Biotechnology, Dalian, China), according to the manufacturer’s instructions. The primer sequences for BACE1 were GGCGGGAGTGGTATTATGA (forward) and TTTCTTGGGCAAACGAAGGT (reverse); primer sequences for ADAM10 were ATGGGAGGTCAGTATGGGAATC (forward) and ACTGCTCTTTTGGCACGCT (reverse). Primers for GAPDH were GAAGGTGAAGGTCGGAGTC (forward) and GAAGATGGTGATGGGATTTC (reverse). The comparative cycle threshold (CT) method was used to calculate the relative gene expression level, with GAPDH as the internal control. Real-time PCR products were analyzed on agarose gel electrophoresis and verified by DNA sequencing.

### RNA Interference

The siRNAs targeting ADAM10 (NM_001110, nucleotides 1715 to 1734, GGGACAAACUUAACAACAAUU, nucleotides 959 to 979, GCUGUGCAGAUCAUUCAGUAU) and SIRT1 (NM_012238, nucleotides 872 to 892, CAGGUCAAGGGAUGGUAUUUA) were obtained from Genepharma Corp. (Shanghai, China) and transiently transfected into HBMEC using Lipofectamine 2000, respectively. The non-silencing siRNA (UUCUCCGAACGUGUCACGUUU) was used as a control [20]. The knockdown effects in the siRNA-transfected HBMEC were analyzed by western blot.

### Immunoprecipitation

HBMEC were washed with ice-cold PBS and lysed with lysis buffer (50 mM Tris, 150 mM NaCl, 2 mM EDTA, 2 mM EGTA, 1% Triton X-100, 1 mM sodium orthovanadate, 25 mM L-glycerophosphate, 1 mM phenylmethylsulfonyl fluoride) containing protease inhibitor cocktail. The cell lysates were centrifuged and the supernatant was collected. The protein content was determined by the Bradford method. A total of 1 mg of protein was incubated with anti-BACE1 antibody (Proteintech, WuHan, China) overnight at 4°C and incubated for 2 h with protein A/G-agarose (Santa Cruz Biotech). The proteins from immune complexes were eluted in SDS sample buffer for western blot analysis.

### Statistical Analysis

All values are presented as mean ± SEM of at least three independent experiments. Statistical significance between two groups was analyzed by Student’s t test. One-way analysis of variance (ANOVA) or two-way ANOVA was used to compare multiple groups. A P value of <0.05 was considered significant.

## Results

### CysC Affects the Releases of Aβ40 and sAPPα from Brain Endothelial Cells

**Fig 1:**
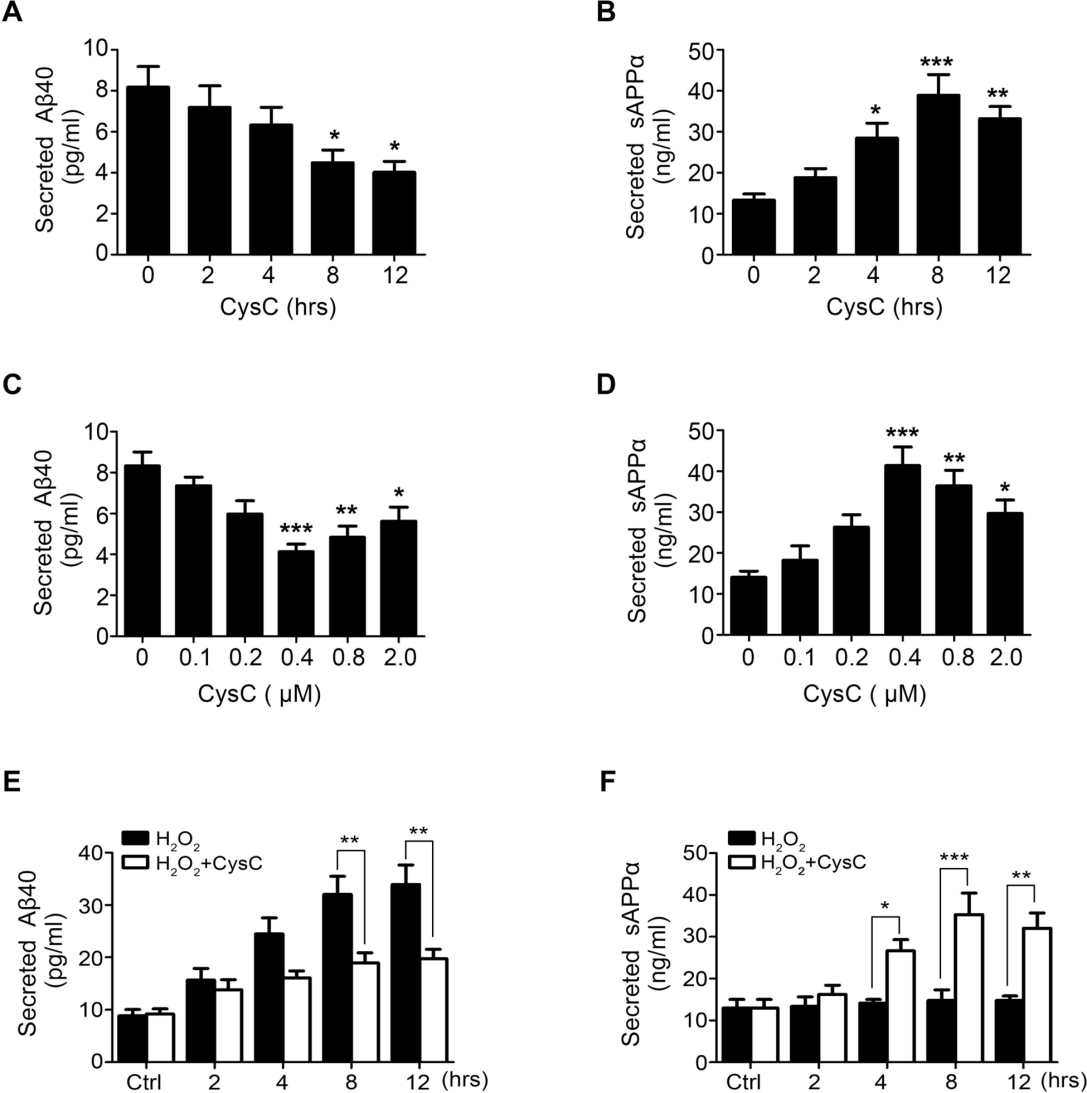
CysC reduces Aβ secretion and promotes sAPPα secretion in HBMEC. (A, B) HBMEC were treated with 0.4 μM CysC for indicated times (0, 2, 4, 8, 12 hr), and the concentrations of Aβ40 (A) and sAPPα (B) levels in the culture medium (supernatant) were determined by ELISA analysis. (C, D) HBMEC were treated with indicated concentrations of CysC for 8 hr. Then the concentrations of Aβ (C) and sAPPα (D) were measured by ELISA. (E, F) HBMEC were pretreated with CysC (0.4 μM) for 4 hr, followed by incubation with 50 μM H2O2 for indicated times (0, 2, 4, 8, 12 hr). Then the concentrations of Aβ (E) and sAPPα (F) were determined by ELISA. All values are presented as mean ± SEM for three independent experiments. Statistical significance was calculated by one-way ANOVA. *, p<0.05; **, p<0.01; ***, p<0.001.

To evaluate the effect of CysC on APP processing in HBMEC, the concentrations of Aβ40 and sAPPα in the culture medium (supernatant) of HBMEC was measured by ELISA. As the physiological concentrations of CysC in the CSF are 0.135–0.693 μM [21], HBMEC were treated with 0.4 μM CysC for indicated times. The results showed that CysC reduced the levels of Aβ40 in the culture medium of HBMEC in a time-dependent manner, with the decrease reaching statistical difference at 8 hr and 12 hr after CysC application (Fig 1A). Meanwhile, the concentration of secreted sAPPα was significantly increased in HBMEC treated with CysC, reaching the peak at 8 hr (Fig 1B). In contrast, secretion of Aβ40 and sAPPα in HBMEC in the absence of CysC showed slightly increase without statistical significance (S1 Fig). The protein expression level of APP in HBMEC was not changed upon CysC treatment (S2 Fig). Then the effect of CysC on HBMEC was examined with different concentrations of CysC. As shown in Fig 1C and 1D, the levels of Aβ40 reduced whereas sAPPα increased in HBMEC treated with increasing concentrations of CysC, both of them reached the peak at 0.4 μM CysC. These results suggested that CysC inhibited endogenous secretion of Aβ40 and promoted endogenous sAPPα secretion in brain endothelial cells.

It has been shown that oxidative stress enhanced Aβ production in HEK293 cells transfected with Swedish mutant form of APP [22,23]. To investigate whether CysC regulates APP processing in HBMEC under oxidative stress condition, HBMEC were treated with H_2_O_2_ (50 μM), which did not affect cell viability (S3 Fig), to mimic the oxidative stress-induced responses, then the concentrations of Aβ40 and sAPPα in the culture medium of HBMEC were measured by ELISA. The secreted Aβ40 increased in a time-dependent manner after H_2_O_2_ treatment, which was effectively abolished by pre-treatment with CysC (Fig 1E). However, the secreted sAPPα in HBMEC was not changed by H_2_O_2_ stimulation (Fig 1F), suggesting H_2_O_2_-induced oxidative stress specifically promoted Aβ40 secretion without any effect on sAPPα. In addition, similar to the findings in Fig 1B, we found the sAPPα secretion were enhanced in a time-dependent manner upon CysC treatment in the presence of H_2_O_2_ (Fig 1F). These results indicated that CysC is able to regulate intracellular APP processing in brain endothelial cells.

### CysC Down-Regulates BACE1 Expression in Brain Endothelial Cells

**Fig 2:**
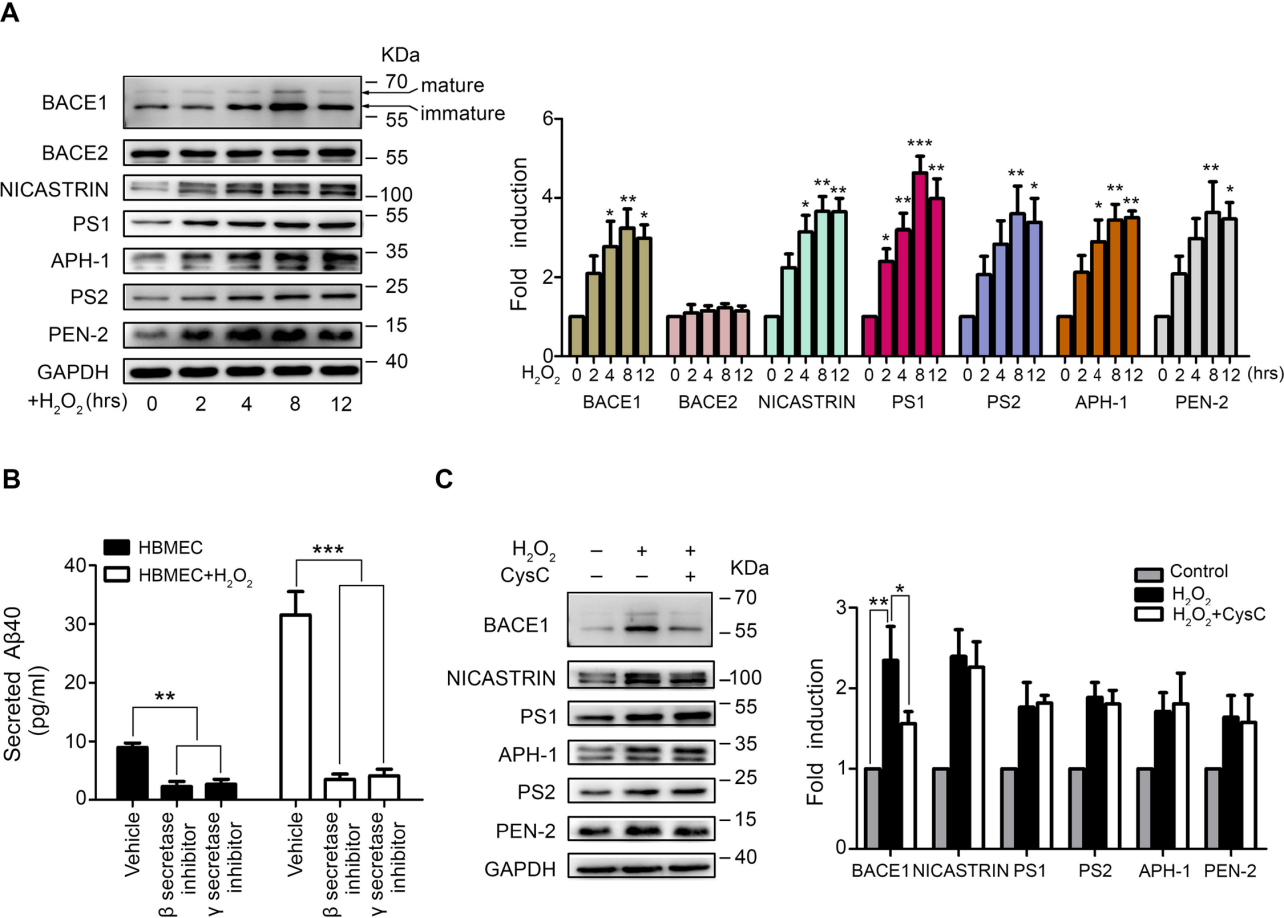
CysC specifically attenuates the increased BACE1 expression induced by H_2_O_2_ in HBMEC. (A) HBMEC were treated with 50 μM H_2_O_2_ for indicated times (0, 2, 4, 8, 12 hr) and then the expression of BACE1, BACE2, NICASTRIN, PS1, APH-1, PS2 and PEN-2 were detected by western blot, with GAPDH served as loading control (left panel). The protein levels were obtained by calculating the band densitometry and normalized to the band intensity of GAPDH, and the values were normalized to control defined as 1 (right panel). Statistical significance was analyzed using one-way ANOVA. *, p<0.05; **, p<0.01; ***, p<0.001. (B) HBMEC were pretreated with β-secretase inhibitor II (1 μM) and γ-secretase inhibitor IX (1 μM) for 1 hr, respectively, with DMSO served as vehicle control. Then the cells were treated with or without H_2_O_2_ (50 μM) for 8 hr. The concentrations of Aβ40 were determined by ELISA assays. Statistical significance was analyzed using one-way ANOVA. **, p<0.01; ***, p<0.001. (C) HBMEC were pretreated with CysC (0.4 μM) for 4 hr followed by incubation with 50 μM H2O2 for 8 hr, and then the expression of BACE1, NICASTRIN, PS1, PS2, APH-1 and PEN-2 were detected by western blot, with GAPDH as the loading control (left panel). The protein levels were obtained by calculating the band densitometry and normalized to the band intensity of GAPDH, and the values were normalized to control (right panel).*, p<0.05; **, p<0.01.

Aβ is generated by a two-step proteolytic cleavage of full-length APP, involving β- and γ-secretases [8,9]. The principal β-secretase is BACE1 [24] and γ-secretase is a multiprotein complex containing presenilin (PS1 or PS2), NICASTRIN, APH-1 and PEN-2 [25]. Here, we found the protein levels of BACE1 (including immature and mature forms), NICASTRIN, PS1, PS2, APH-1 and PEN-2 were significantly increased in HBMEC treated with H_2_O_2_ (Fig 2A). In contrast, BACE2, a β-secretase homolog cleaves APP within the Aβ region and is not involved in Aβ40 generation [26], was not affected (Fig 2A). Both β-secretase Inhibitor II and γ-secretase Inhibitor IX could significantly abrogated the increased Aβ40 secretion in H_2_O_2_-treated HBMEC as well as in normal HBMEC (Fig 2B). These results prompted us to test whether the effect of CysC to prevent Aβ40 secretion is achieved by affecting the levels of β- and γ-secretases. The results showed that CysC treatment attenuated the H_2_O_2_-induced BACE1 increase in HBMEC, without affecting the expression levels of γ-secretases including NICASTRIN, PS1, PS2, APH-1 and PEN-2 (Fig 2C). These data indicated that CysC specifically downregulates intracellular β-secretase BACE1 to prevent Aβ production in HBMEC under oxidative stress condition.

### CysC Promotes Proteasomal Degradation of BACE1 in Brain Endothelial Cells

**Fig 3:**
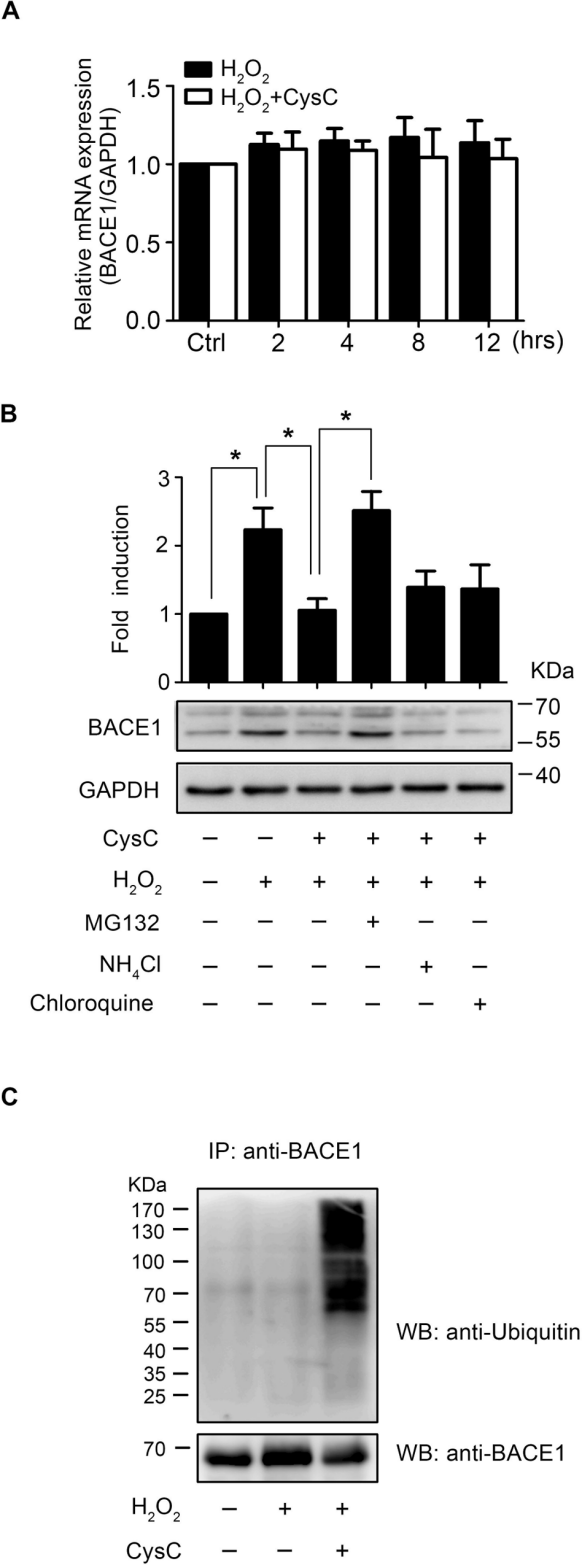
CysC enhances proteasomal degradation of BACE1 in HBMEC. (A) HBMEC were treated with 50 μM H_2_O_2_ for indicated times in the absence or presence of CysC (0.4 μM) and the mRNA levels of BACE1 were analyzed by real-time RT-PCR, with GADPH as internal control. Data were normalized to control. (B) HBMEC were pretreated with CysC (0.4 μM) for 4 hr and then the cells were incubated with MG132 (5 μM), chloroquine (100 μM) or NH4Cl (20 mM) for 1 hr, followed by incubation with H2O2 (50 μM) for 8 hr. Then the protein levels of BACE1 were detected by western blot. GAPDH was used as the loading control. Statistical significance was calculated using two-way ANOVA. *, p<0.05. (C) HBMEC were pretreated with or without CysC (0.4 μM) for 4 hr followed by incubation with H_2_O_2_ (50 μM) for 8 hr. Total cells lysates were immunoprecipitated with BACE1 antibody and then the ubiquitinated BACE1 was detected by western blot with anti-ubiquitin antibody. Representative results from 3 independent experiments were presented.

To clarify the mechanism of CysC-triggered BACE1 reduction in H_2_O_2_-induced HBMEC, real-time RT-PCR was performed to analyze the mRNA level of BACE1. The results showed that CysC treatment did not affect BACE1 mRNA expression in H_2_O_2_-treated HBMEC (Fig 3A), suggesting the decrease of BACE1 protein induced by CysC was caused by degradation of intracellular BACE1 protein. It has been revealed that BACE1 can be degraded via the ubiquitin-proteasome pathway [27] as well as the lysosomal pathway [28]. Thus, to determine the involved pathway for the CysC-induced BACE1 reduction in H_2_O_2_-treated HBMEC, the cells were pre-incubated with proteasome inhibitor MG132 and lysosomal inhibitors including chloroquine and NH_4_Cl, lysed and subjected to western blot to measure BACE1 levels. We found that MG132 treatment significantly attenuated the CysC-induced BACE1 reduction in H_2_O_2_-treated HBMEC, whereas chloroquine and NH_4_Cl had no such effect (Fig 3B), suggesting CysC-induced BACE1 reduction was caused by ubiquitin-proteasome pathway. Then, H_2_O_2_-treated HBMEC in the absence or presence of CysC were subjected to immunoprecipitation assay with BACE1 antibody, and the precipitates were examined by western blot using ubiquitin antibody (Fig 3C). We found a significant high level of ubiquitinated BACE1 in H_2_O_2_-treated HBMEC pre-incubated with CysC compared to HBMEC exposed to H_2_O_2_ alone. These results demonstrated that CysC promotes BACE1 degradation through the ubiquitin/proteasome pathway in brain endothelial cells under oxidative stress conditions.

### CysC-Induced sAPPα Secretion Is Associated with α-Secretase ADAM10 in Brain Endothelial Cells

**Fig 4:**
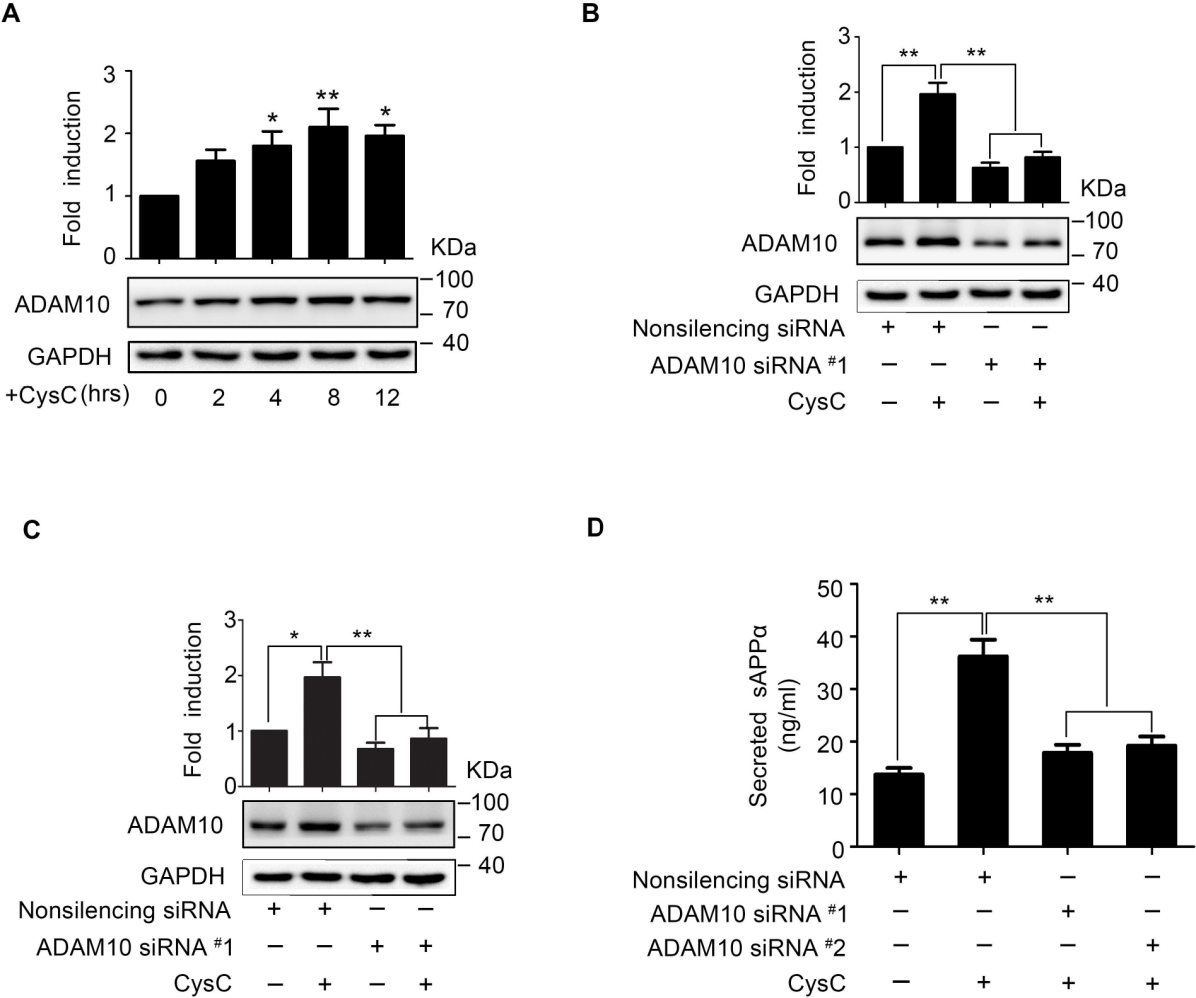
CysC-induced sAPPα secretion is mediated by upregulation of ADAM10 in HBMEC. (A) HBMEC were treated with CysC (0.4 μM) for 0, 2, 4, 8, 12 hr, respectively, and then the protein levels of ADAM10 were detected by western blot, with GAPDH as loading control. The band densitometry were measured and normalized to GAPDH, and the values were normalized to control. Statistical significance was analyzed with one-way ANOVA. *, p<0.05; **, p<0.01. (B, C) HBMEC were transiently transfected with ADAM10 siRNA#1(B) and ADAM10 siRNA#2 (C), with non-silencing siRNA as control. 48 hr later, the cells were treated with CysC (0.4 μM) for 8 hr. Then ADAM10 expression was analyzed by western blot, with GAPDH as loading control. The band densitometry were measured and normalized to GAPDH, and the values were normalized to control. Statistical significance was analyzed with one-way ANOVA. *, p<0.05; **, p<0.01. (D) The HBMEC transfected with ADAM10 siRNA were incubated with CysC (0.4 μM) for 8 hr, with non-silencing siRNA as a control. Then the secreted sAPPα were determined by ELISA assay. The values are means ± SEM of three independent experiments. **, P<0.01.

It is known that sAPPα is the non-amyloidogenic product of APP cleaved by α-secretases. ADAM10, a transmembrane metalloprotease, has been demonstrated as the major α-secretase producing sAPPα [29,30]. To determine the mechanism of increased sAPPα secretion induced by CysC (Fig 1B, 1D and 1F), the expression of ADAM10 in HBMEC treated with CysC was assessed by western blot. We found the protein levels of ADAM10 were significantly elevated in HBMEC upon CysC treatment, reaching the peak at 8 hr after treatment (Fig 4A). Then siRNA-mediated RNA interference was used to knockdown ADAM10 in HBMEC (Fig 4B and 4C). The ADAM10 siRNA were synthesized and transiently transfected into HBMEC, and the knockdown effect was evaluated by western blot. The results showed that ADAM10 in HBMEC was reduced by two different siRNA recognizing ADAM10 (Fig 4B and 4C) compared to the non-silencing siRNA control. Also, we found the CysC-induced ADAM10 upregulation in HBMEC was effectively abolished by ADAM10 knockdown (Fig 4B and 4C). Then, HBMEC with ADAM10 knockdown were incubated with or without CysC followed by measurement of sAPPα secretion in the culture medium. As shown in Fig 4D, ADAM10 knockdown in HBMEC significantly prevented the CysC-induced sAPPα secretion compared to the control. These results indicated that ADAM10 is essential for the CysC-promoted sAPPα secretion in brain endothelial cells.

### CysC Upregulates *ADAM10* mRNA via SIRT1 to Promote sAPPα Secretion in Brain Endothelial Cells

**Fig 5:**
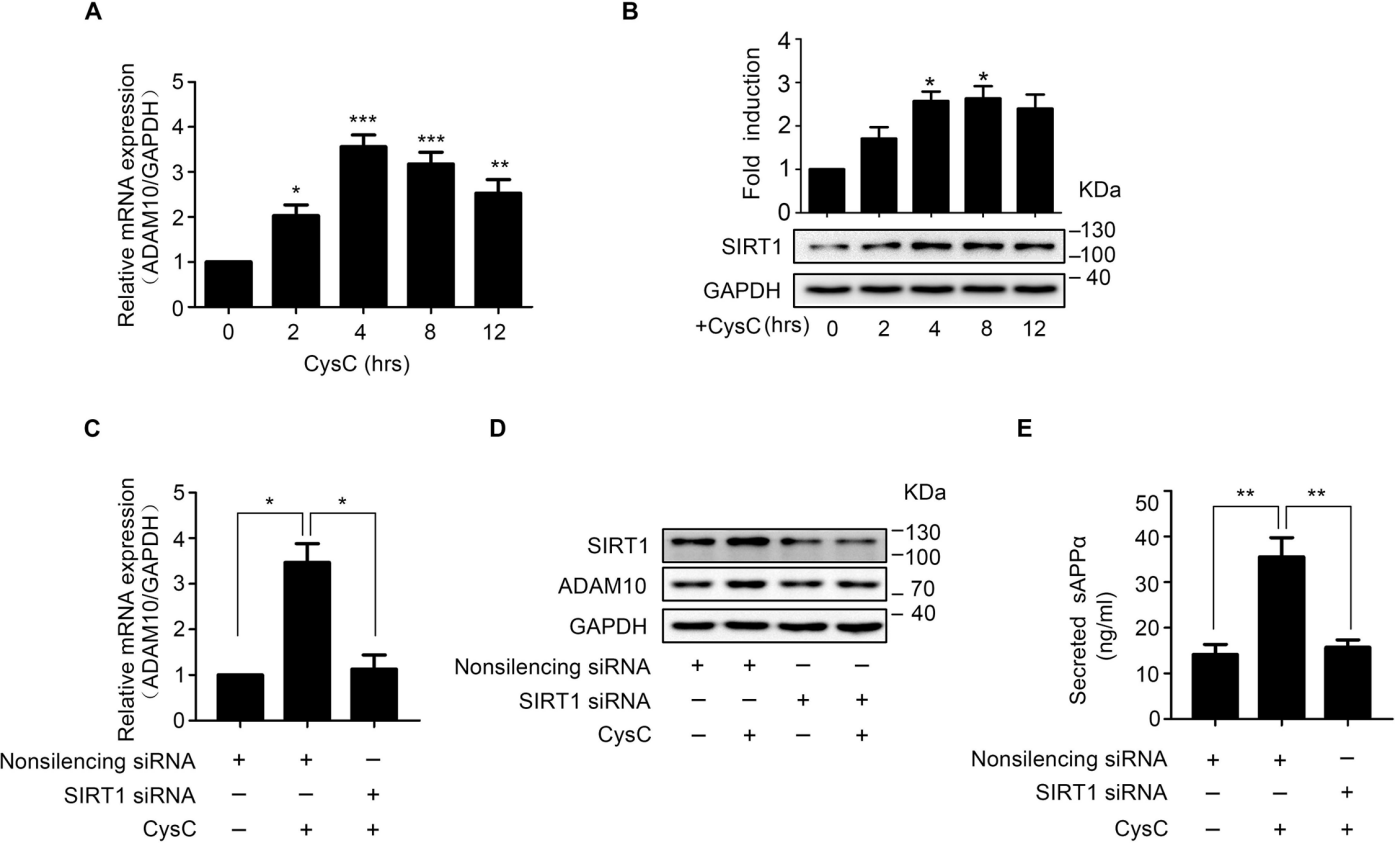
CysC enhances SIRT1 expression to upregulate ADAM10 mRNA levels, leading to increased sAPPα secretion. (A) HBMEC were treated with CysC for indicated times (0, 2, 4, 8, 12 hr), and the mRNA expressions of ADAM10 were analyzed by real-time RT-PCR, with GADPH as internal control. Data were normalized to control. Statistical significance was analyzed using one-way ANOVA. *, p<0.05; **, p<0.01; ***, p<0.001. (B) HBMEC were incubated with CysC for indicated times (0, 2, 4, 8, 12 hr), then the protein levels of SIRT1 were detected by western blot with GAPDH as the loading control. The band densitometry were measured and normalized to GAPDH, and the values were normalized to control. Statistical significance was analyzed with one-way ANOVA. *, p<0.05. (C-E) HBMEC were transiently transfected with SIRT1 siRNA, with non-silencing siRNA as a control. 48 hr later, the cells were treated with CysC for 8 hr, and the mRNA (C) and protein (D) levels of ADAM10, as well as sAPPα secretion (E) were determined. Statistical significance was calculated with one-way ANOVA.*, p<0.05; **, p<0.01.

To further dissect the mechanism of increased ADAM10 protein expression induced by CysC, the mRNA levels of ADAM10 were analyzed by real-time RT-PCR. The results showed that ADAM10 mRNA were significantly increased in HBMEC incubated with CysC (Fig 5A). The ADAM10 mRNA increased to reach statistical significance at 2 hr time point after CysC treatment, which is earlier than the 8 hr time point in ADAM10 protein changes (Fig 4A). Also, the peak of ADAM10 mRNA increase, which was at 4 hr after CysC treatment, is earlier than the peak time (8 hr) of ADAM10 protein changes (Fig 4A). Thus, the increased ADAM10 mRNA occurred earlier than the changes of ADAM10 protein levels in HBMEC upon CysC treatment.

In neuroblastoma N2a cells expressing human APP Swedish mutant, overexpression of the SIRT1 gene increased ADAM10 protein expression [31]. These prompt us to test whether the CysC-induced ADAM10 upregulation is caused by SIRT1. Western Blot results showed that CysC significantly promoted protein levels of SIRT1 in HBMEC in a time-dependent manner (Fig 5B). To verify that CysC-induced ADAM10 upregulation is mediated by SIRT1, siRNA targeting to SIRT1 were transfected into HBMEC to reduce SIRT1 protein levels in HBMEC. The subsequent results showed that the mRNA and protein levels of ADAM10 were significantly attenuated in HBMEC with silenced SIRT1 in response to CysC treatment (Fig 5C and 5D). In other words, SIRT1 knockdown effectively prevented the CysC-induced ADAM10 upregulation in HBMEC. Moreover, CysC failed to promote the secretion of sAPPα in HBMEC with silenced SIRT1 compared to the non-silencing siRNA control (Fig 5E). These results demonstrated that CysC upregulates ADAM10 at transcriptional level, mediated by SIRT1 signaling, to facilitate sAPPα secretion in brain endothelial cells.

## Discussion

Aβ is a proteolytic product of sequential cleavage of APP protein by secretases. In AD, pathological Aβ deposition in the brain forms senile plaques and Aβ accumulation in cerebral vessel wall produces CAA, both of which are the characteristic lesions of AD [1,3]. Aβ40 and Aβ42 are the predominant Aβ species with quite similar sequences, and the only difference between them is an extra isoleucine and analanine at the C-terminus of Aβ42. Aβ42 is more amyloidogenic than Aβ40, and is deposited earlier than Aβ40 in the brain parenchyma in AD patients. Aβ42 is the major isoform in the amyloid plaque in the brain of AD, whereas Aβ40 aggregates are predominantly found in the vascular wall in CAA [1,3,9]. Modulating the processing of APP has important implications for intervention strategies to prevent Aβ deposition in AD. In this study, we found CysC reduced Aβ40 secretion via proteasomal degradation of β-secretase BACE1 in brain endothelial cells. Meanwhile, CysC promoted sAPPα release by transcriptional upregulation of α-secretase ADAM10. Thus CysC is able to shift the balance of APP processing from the amyloidogenic β-cleavage to non-amyloidogenic α-cleavage, producing less Aβ40 and more sAPPα in brain endothelial cells.

Several compounds (synthetic or natural) have been shown to switch APP processing to non-amyloidogenic route. L-3-n-butylphthalide (L-NBP), an extract from seeds of Apium graveolens Linn (Chinese celery), promotes sAPPα release and reduces Aβ generation in neuroblastoma cells after 24-hr treatment [32]. Neuroprotectin D1 (NPD1), a stereoselective mediator derived from the omega-3 essential fatty acid docosahexaenoic acid (DHA), suppresses Aβ42 peptide shedding and upregulates intracellular sAPPα expression in neuronal-glial co-cultured cells over-expressing APP_sw_ (Swedish double mutation with K595N and M596L) after 48-hr treatment [33]. Carbachol, a muscarinic receptor agonist, caused an increase of sAPPα secretion in teratocarcinoma-derived neurons, as well as a decrease in Aβ production in the medium [34]. To our knowledge, CysC is the first naturally occurring protein described to direct APP metabolism from the amyloidogenic pathway towards non-amyloidogenic pathway. In the brain, CysC was found to be present in neurons and microglial cells [35,36] (but not in astrocytes [36]), whereas the expression of CysC in brain endothelial cells was undetectable (S4 Fig), these support the scenario that neuronal cell-derived extracellular CysC acts directly on brain endothelial cells via paracrine mechanism to affect endothelial processing of APP.

The association of CysC with brain disorders has been reported. Hereditary CysC amyloid angiopathy (HCCAA), also called hereditary cerebral hemorrhage with amyloidosis of Icelandic type, is an autosomal dominant form of CAA. The amyloid deposition in the vessel walls caused fatal brain hemorrhage in normotensive young adults because of a Leu68Gln mutation in CysC [37]. Also, variant B of CysC, containing a single mutation A25T, is associated with age-related macular degeneration (AMD) and AD [38]. A most recent study indicated that variant B of CysC is inefficiently secreted which impairs its protective effect against Aβ aggregation [39]. CysC was identified to interact with APP protein within the Aβ region [17]. The influence of CysC on Aβ aggregation was studied and the results showed CysC could inhibit formation of Aβ fibrils [17,40,41] and Aβ oligomers [18,19]. These studies suggested CysC may exert protective effect against Aβ deposition in AD. In this study, we found endogenous Aβ production was reduced upon CysC administration due to proteasomal degradation of β-secretase BACE1 in brain endothelial cells. Our findings thus unveil a previously unrecognized effect of CysC to reduce Aβ secretion. In addition, CysC stimulates release of sAPPα in brain endothelial cells. It has been demonstrated that sAPPα has protective properties against glucose deprivation, glutamate neurotoxicity [11] and Aβ-induced oxidative injury [42] in cultured neurons as well as ischemic injury of rat hippocampus *in vivo* [43]. Thus, the ability of CysC to reduce Aβ secretion and promote sAPPα release indicated its protective function, which is in line with the neuroprotective effect of CysC in AD [14].

When the concentration reached to 0.4 μM, CysC significantly inhibited Aβ40 secretion (Fig 1C) and promoted sAPPα release (Fig 1D) in HBMEC. Interestingly, with the increase of concentration to 0.8 and 2.0 μM, the effect of CysC on Aβ and sAPPα secretion was less prominent than at 0.4 μM (Fig 1C and 1D). These indicated the effect of CysC on APP processing is strictly associated with its concentrations and is saturated at 0.4 μM. Similar to our findings, Martinez-Vargas et al. found that lower-dose (3.5 fmoles) injection of CysC into the rat brain with traumatic injury reduced bleeding and mortality, whereas high doses (35 and 175 fmoles) had little effect on bleeding and mortality [44]. Based on these results, we recommend to be more cautious regarding the concentration of CysC used in the evaluation of the effect of exogenously applied CysC.

In Pawlik et al.’s pioneer study on CysC, transgenic mice expressing either wild-type or the Leu68Gln variant CysC genes were generated [45]. They found that the CysC transgenic mice are fertile and their appearance are indistinguishable from littermate controls. Those mice showed no obvious behavioral defects, without any gross pathological or histopathological abnormalities up to 6 month of age. Similar levels of Aβ40 and Aβ42 were found in the brain homogenates of CysC transgenic mice compared to littermate controls [45], which appeared inconsistent with our study. This discrepancy may reflect the different manipulations in Pawlik M et al.’s and our study. The acute application of recombinant CysC to treat brain endothelial cells in our study revealed that CysC caused a rapid reduction of Aβ40 secretion within a short time window, from 4 hr to 12 hr after application of protein CysC. In contrast, the *in vivo* overexpression of CysC in 3–8 month transgenic mice has little effect on brain Aβ level [45] are likely due to developmental compensation that could mask the acute effect of CysC during the 3–8 month development. In addition, we used brain endothelial cells to analyze its Aβ secretion in response to CysC treatment, which is different from Pawlik M et al.’s study in which they measured the Aβ level in the whole brain homogenates [45].

So far the effects of CysC on brain Aβ levels were more complicated than expected. It has been reported that overexpression of CysC reduced plaque loads without affecting soluble brain Aβ levels in mice [40,41]. Surprisingly, Sun et al. found that both the soluble Aβ levels and plaque load were reduced in CysC knockout mice due to cathepsin B-induced Aβ degradation [46]. In this study, our results showed that application of recombinant CysC protein decreased Aβ40 secretion in brain endothelial cells. It is difficult to reconcile these puzzling findings of CysC with current understandings of CysC. Thus further study is necessary to clarify the effect of CysC on Aβ metabolism as well as the underlying mechanism.

BACE1 is the major β-secretase enzyme for the production of Aβ from proteolytic processing of APP [8,9]. We found the increased BACE1 in brain endothelial cells upon H_2_O_2_ stimulation was significantly attenuated by CysC (Fig 2C). In contrast, the H_2_O_2_–induced increase of γ-secretases (including NICASTRIN, PS1, PS2, APH-1 and PEN2) remained unchanged after CysC application (Fig 2C). These suggested that CysC specifically down-regulates BACE1 expression in brain endothelial cells. Moreover, we found CysC could effectively reduce H_2_O_2_-induced Aβ secretion (Fig 1E) though the γ-secretases remained increased. Thus in this context, we concluded that β-secretase BACE1 is the critical enzyme in the production of Aβ from APP in brain endothelial cells. This is compatible with previous findings that BACE1 processing is the key step for Aβ generation in the brain [47,48].

The expression of BACE1 was modulated by transcriptional and post-transcriptional controls [49]. Here we found the protein (Fig 2A), but not mRNA (Fig 3A), levels of BACE1 were elevated in brain endothelial cells upon H_2_O_2_ stimulation, which was significantly attenuated by CysC (Fig 2C), suggesting CysC affects BACE1 expression by post-transcriptional regulation. Our further results revealed that CysC promotes proteasomal degradation of BACE1 (Fig 3B and 3C) which points out post-translational modification of BACE1 initiated by CysC. Previous studies have documented the transcriptional control of BACE1, whereas the post-transcriptional regulation of BACE1 was reported until recently. It was shown that peroxisome proliferator-activated receptor-γ coactivator 1 (PGC)-1α and E3-ligase CHIP promotes BACE1 degradation via proteasomal pathway [50,51]. Regarding the CysC-induced BACE1 proteasomal degradation in brain endothelial cells, whether it is dependent on PGC-1α or CHIP, or an alternative unrecognized signaling pathway, remains to be determined in future studies.

In summary, our study demonstrated that CysC reduces Aβ40 secretion and facilitates sAPPα secretion in brain endothelial cells. The inhibition of Aβ40 secretion is caused by the CysC-induced degradation of BACE1 through the ubiquitin/proteasome pathway, whereas the enhanced sAPPα secretion is due to increased expression of ADAM10 mediated by SIRT1. Our findings point out the novel role of CysC in APP processing which suggests a potential therapeutic application in AD.

## Supporting Information

S1 Fig Aβ40 and sAPPα secretion in HBMEC. (A) HBMEC were cultured for indicated times, and the concentrations of Aβ40 levels in the culture medium (supernatant) were determined by ELISA assay. (B) HBMEC were cultured for indicated times with or without Cystatin C (CysC, 0.4 μM), and the concentrations of sAPPα in the culture medium (supernatant) were determined by ELISA assay. *, P <0.05; **, P <0.01.
(EPS)

S2 Fig APP expression is not altered in HBMEC treated with CysC. HBMEC were treated with CysC (0.4 μM) for indicated times and then the protein levels of APP were detected by western blot, with GAPDH served as the loading control. The protein levels were obtained by calculating the band densitometry and normalized to the band intensity of GAPDH, and the values were normalized to control defined as 1. Presented results are from three independent experiments.
(EPS)

S3 Fig Effect of H_2_O_2_ on cell viability of HBMEC. HBMECs were treated with 50 μM H_2_O_2_ for 8 hr, and the cells were stained with 2-(2-methoxy-4-nitrophenyl)-3-(4-nitrophenyl)-5-(2,4-disulfophenyl)-2H-tetrazolium,monosodium salt (WST) to determine the cell viability. Data are presented as the percentage of control cells. All the values are presented as the mean ± SEM from three independent experiments.
(EPS)

S4 Fig Cystatin C is undetectable in HBMEC. HBMEC and 293T cells were lysed with RIPA buffer, and then the protein expression of Cystatin C was detected by western blot, with GAPDH as the loading control. Presented results are from at least three independent experiments.
(EPS)

## Abbreviations

CysC: Cystatin C
HBMEC: human brain microvascular endothelial cells
AD: Alzheimer’s disease
CAA: cerebral amyloid angiopathy
H_2_O_2_: hydrogen peroxide
APP: amyloid protein precursor
Aβ: amyloid-β
sAPPα: soluble APPα
BACE1: beta-site APP cleaving enzyme 1
BACE2: beta-site APP cleaving enzyme 2
ADAM: a disintegrin and metalloprotease domain
PS: presenilins
APH-1: anterior pharynx-defective-1
PEN-2: presenilin enhancer-2
SIRT1: Sirtuin type 1

## References

[1] Scheltens P, Blennow K, Breteler MM, de Strooper B, Frisoni GB, Salloway S, et al (2016) Alzheimer's disease. Lancet. 10.1016/S0140-6736(15)01124-1 26921134

[2] Attems J, Yamaguchi H, Saido TC, Thal DR (2010) Capillary CAA and perivascular Abeta-deposition: two distinct features of Alzheimer's disease pathology. J Neurol Sci 299: 155–162. 10.1016/j.jns.2010.08.030 20850138

[3] Greenberg SM, Gurol ME, Rosand J, Smith EE (2004) Amyloid angiopathy-related vascular cognitive impairment. Stroke 35: 2616–2619. 15459438 10.1161/01.STR.0000143224.36527.44

[4] Deane R, Wu Z, Sagare A, Davis J, Du Yan S, Hamm k, et al (2004) LRP/amyloid beta-peptide interaction mediates differential brain efflux of Abeta isoforms. Neuron 43: 333–344. 15294142 10.1016/j.neuron.2004.07.017

[5] Tarasoff-Conway JM, Carare RO, Osorio RS, Glodzik L, Butler T, Fieremans E, et al (2015) Clearance systems in the brain-implications for Alzheimer disease. Nat Rev Neurol 11: 457–470. 10.1038/nrneurol.2015.119 26195256 PMC4694579

[6] Austin SA, Santhanam AV, Katusic ZS (2010) Endothelial nitric oxide modulates expression and processing of amyloid precursor protein. Circ Res 107: 1498–1502. 10.1161/CIRCRESAHA.110.233080 21127294 PMC3064266

[7] Kitazume S, Tachida Y, Kato M, Yamaguchi Y, Honda T, Hashimoto Y, et al (2010) Brain endothelial cells produce amyloid {beta} from amyloid precursor protein 770 and preferentially secrete the O-glycosylated form. J Biol Chem 285: 40097–40103. 10.1074/jbc.M110.144626 20952385 PMC3000992

[8] Thinakaran G, Koo EH (2008) Amyloid precursor protein trafficking, processing, and function. J Biol Chem 283: 29615–29619. 10.1074/jbc.R800019200 18650430 PMC2573065

[9] Zhang YW, Thompson R, Zhang H, Xu H (2011) APP processing in Alzheimer's disease. Mol Brain 4: 3 10.1186/1756-6606-4-3 21214928 PMC3022812

[10] Walsh DM, Selkoe DJ (2007) A beta oligomers—a decade of discovery. J Neurochem 101: 1172–1184. 17286590 10.1111/j.1471-4159.2006.04426.x

[11] Mattson MP, Cheng B, Culwell AR, Esch FS, Lieberburg I, Rydel RE (1993) Evidence for excitoprotective and intraneuronal calcium-regulating roles for secreted forms of the beta-amyloid precursor protein. Neuron 10: 243–254. 8094963 10.1016/0896-6273(93)90315-i

[12] Ring S, Weyer SW, Kilian SB, Waldron E, Pietrzik CU, Filippov MA, et al (2007) The secreted beta-amyloid precursor protein ectodomain APPs alpha is sufficient to rescue the anatomical, behavioral, and electrophysiological abnormalities of APP-deficient mice. J Neurosci 27: 7817–7826. 17634375 10.1523/JNEUROSCI.1026-07.2007 PMC6672885

[13] Turk V, Stoka V, Turk D (2008) Cystatins: biochemical and structural properties, and medical relevance. Front Biosci 13: 5406–5420. 18508595 10.2741/3089

[14] Gauthier S, Kaur G, Mi W, Tizon B, Levy E (2011) Protective mechanisms by cystatin C in neurodegenerative diseases. Front Biosci (Schol Ed) 3: 541–554. 21196395 10.2741/s170 PMC3038625

[15] Hansson SF, Andreasson U, Wall M, Skoog I, Andreasen N, Wallin A, et al (2009) Reduced levels of amyloid-beta-binding proteins in cerebrospinal fluid from Alzheimer's disease patients. J Alzheimers Dis 16: 389–397. 10.3233/JAD-2009-0966 19221428

[16] Bai Y, Markham K, Chen F, Weerasekera R, Watts J, Horne P, et al (2008) The in vivo brain interactome of the amyloid precursor protein. Mol Cell Proteomics 7: 15–34. 17934213 10.1074/mcp.M700077-MCP200

[17] Sastre M, Calero M, Pawlik M, Mathews PM, Kumar A, Danilov V, et al (2004) Binding of cystatin C to Alzheimer's amyloid beta inhibits in vitro amyloid fibril formation. Neurobiol Aging 25: 1033–1043. 15212828 10.1016/j.neurobiolaging.2003.11.006

[18] Selenica ML, Wang X, Ostergaard-Pedersen L, Westlind-Danielsson A, Grubb A (2007) Cystatin C reduces the in vitro formation of soluble Abeta1-42 oligomers and protofibrils. Scand J Clin Lab Invest 67: 179–190. 17365997 10.1080/00365510601009738

[19] Tizon B, Ribe EM, Mi W, Troy CM, Levy E (2010) Cystatin C protects neuronal cells from amyloid-beta-induced toxicity. J Alzheimers Dis 19: 885–894. 10.3233/JAD-2010-1291 20157244 PMC2889175

[20] Liu Y, Chu A, Chakroun I, Islam U, Blais A (2010) Cooperation between myogenic regulatory factors and SIX family transcription factors is important for myoblast differentiation. Nucleic Acids Res 38: 6857–6871. 10.1093/nar/gkq585 20601407 PMC2978361

[21] Yamada T, Mukaiyama I, Miyake N, Igari J (2002) Measurement of cystatin C in cerebrospinal fluid. Rinsho Byori 50: 613–617. 12166082

[22] Shen C, Chen Y, Liu H, Zhang K, Zhang T, Lin A, et al (2008) Hydrogen peroxide promotes Abeta production through JNK-dependent activation of gamma-secretase. J Biol Chem 283: 17721–17730. 10.1074/jbc.M800013200 18436531 PMC2427353

[23] Tong Y, Zhou W, Fung V, Christensen MA, Qing H, Sun X, et al (2005) Oxidative stress potentiates BACE1 gene expression and Abeta generation. J Neural Transm (Vienna) 112: 455–469. 15614428 10.1007/s00702-004-0255-3

[24] Cai H, Wang Y, McCarthy D, Wen H, Borchelt DR, Price DL, et al (2001) BACE1 is the major beta-secretase for generation of Abeta peptides by neurons. Nat Neurosci 4: 233–234. 11224536 10.1038/85064

[25] De Strooper B (2003) Aph-1, Pen-2, and Nicastrin with Presenilin generate an active gamma-Secretase complex. Neuron 38: 9–12. 12691659 10.1016/s0896-6273(03)00205-8

[26] Farzan M, Schnitzler CE, Vasilieva N, Leung D, Choe H (2000) BACE2, a beta -secretase homolog, cleaves at the beta site and within the amyloid-beta region of the amyloid-beta precursor protein. Proc Natl Acad Sci U S A 97: 9712–9717. 10931940 10.1073/pnas.160115697 PMC16930

[27] Qing H, Zhou W, Christensen MA, Sun X, Tong Y, Song W (2004) Degradation of BACE by the ubiquitin-proteasome pathway. FASEB J 18: 1571–1573. 15289451 10.1096/fj.04-1994fje

[28] Koh YH, von Arnim CA, Hyman BT, Tanzi RE, Tesco G (2005) BACE is degraded via the lysosomal pathway. J Biol Chem 280: 32499–32504. 16033761 10.1074/jbc.M506199200

[29] Jorissen E, Prox J, Bernreuther C, Weber S, Schwanbeck R, Serneels L, et al (2010) The disintegrin/metalloproteinase ADAM10 is essential for the establishment of the brain cortex. J Neurosci 30: 4833–4844. 10.1523/JNEUROSCI.5221-09.2010 20371803 PMC2921981

[30] Kuhn PH, Wang H, Dislich B, Colombo A, Zeitschel U, Ellwart JW, et al (2010) ADAM10 is the physiologically relevant, constitutive alpha-secretase of the amyloid precursor protein in primary neurons. EMBO J 29: 3020–3032. 10.1038/emboj.2010.167 20676056 PMC2944055

[31] Lee HR, Shin HK, Park SY, Kim HY, Lee WS, Rhim BY, et al (2014) Cilostazol suppresses beta-amyloid production by activating a disintegrin and metalloproteinase 10 via the upregulation of SIRT1-coupled retinoic acid receptor-beta. J Neurosci Res 92: 1581–1590. 10.1002/jnr.23421 24903973

[32] Peng Y, Hu Y, Xu S, Feng N, Wang L, Wang X (2011) L-3-n-butylphthalide regulates amyloid precursor protein processing by PKC and MAPK pathways in SK-N-SH cells over-expressing wild type human APP695. Neurosci Lett 487: 211–216. 10.1016/j.neulet.2010.10.025 20969923

[33] Zhao Y, Calon F, Julien C, Winkler JW, Petasis NA, Lukiw WJ, et al (2011) Docosahexaenoic acid-derived neuroprotectin D1 induces neuronal survival via secretase- and PPARgamma-mediated mechanisms in Alzheimer's disease models. PLoS One 6: e15816 10.1371/journal.pone.0015816 21246057 PMC3016440

[34] Wolf BA, Wertkin AM, Jolly YC, Yasuda RP, Wolfe BB, Konrad RJ, et al (1995) Muscarinic regulation of Alzheimer's disease amyloid precursor protein secretion and amyloid beta-protein production in human neuronal NT2N cells. J Biol Chem 270: 4916–4922. 7876266 10.1074/jbc.270.9.4916

[35] Deng A, Irizarry MC, Nitsch RM, Growdon JH, Rebeck GW (2001) Elevation of cystatin C in susceptible neurons in Alzheimer's disease. Am J Pathol 159: 1061–1068. 11549598 10.1016/S0002-9440(10)61781-6 PMC1850464

[36] Lukasiuk K, Pirttila TJ, Pitkanen A (2002) Upregulation of cystatin C expression in the rat hippocampus during epileptogenesis in the amygdala stimulation model of temporal lobe epilepsy. Epilepsia 43 Suppl 5: 137–145. 12121309 10.1046/j.1528-1157.43.s.5.20.x

[37] Palsdottir A, Snorradottir AO, Thorsteinsson L (2006) Hereditary cystatin C amyloid angiopathy: genetic, clinical, and pathological aspects. Brain Pathol 16: 55–59. 16612982 10.1111/j.1750-3639.2006.tb00561.x PMC8095917

[38] Butler JM, Sharif U, Ali M, McKibbin M, Thompson JP, Gale R, et al (2015) A missense variant in CST3 exerts a recessive effect on susceptibility to age-related macular degeneration resembling its association with Alzheimer's disease. Hum Genet 134: 705–715. 10.1007/s00439-015-1552-7 25893795 PMC4460273

[39] Sant'Anna R, Navarro S, Ventura S, Paraoan L, Foguel D (2016) Amyloid properties of the leader peptide of variant B cystatin C: implications for Alzheimer and macular degeneration. FEBS Lett 590: 644–654. 10.1002/1873-3468.12093 26865059

[40] Kaeser SA, Herzig MC, Coomaraswamy J, Kilger E, Selenica ML, Winkler DT, et al (2007) Cystatin C modulates cerebral beta-amyloidosis. Nat Genet 39: 1437–1439. 18026102 10.1038/ng.2007.23

[41] Mi W, Pawlik M, Sastre M, Jung SS, Radvinsky DS, Klein AM, et al (2007) Cystatin C inhibits amyloid-beta deposition in Alzheimer's disease mouse models. Nat Genet 39: 1440–1442. 18026100 10.1038/ng.2007.29

[42] Goodman Y, Mattson MP (1994) Secreted forms of beta-amyloid precursor protein protect hippocampal neurons against amyloid beta-peptide-induced oxidative injury. Exp Neurol 128: 1–12. 8070512 10.1006/exnr.1994.1107

[43] Smith-Swintosky VL, Pettigrew LC, Craddock SD, Culwell AR, Rydel RE, Mattson MP (1994) Secreted forms of beta-amyloid precursor protein protect against ischemic brain injury. J Neurochem 63: 781–784. 8035204 10.1046/j.1471-4159.1994.63020781.x

[44] Martinez-Vargas M, Soto-Nunez M, Tabla-Ramon E, Solis B, Gonzalez-Rivera R, Perez-Arredondo A, et al (2014) Cystatin C has a dual role in post-traumatic brain injury recovery. Int J Mol Sci 15: 5807–5820. 10.3390/ijms15045807 24714089 PMC4013597

[45] Pawlik M, Sastre M, Calero M, Mathews PM, Schmidt SD, Nixon RA, et al (2004) Overexpression of human cystatin C in transgenic mice does not affect levels of endogenous brain amyloid Beta Peptide. J Mol Neurosci 22: 13–18. 14742906 10.1385/JMN:22:1-2:13

[46] Sun B, Zhou Y, Halabisky B, Lo I, Cho SH, Mueller-Steiner S, et al (2008) Cystatin C-cathepsin B axis regulates amyloid beta levels and associated neuronal deficits in an animal model of Alzheimer's disease. Neuron 60: 247–257. 10.1016/j.neuron.2008.10.001 18957217 PMC2755563

[47] Vassar R, Bennett BD, Babu-Khan S, Kahn S, Mendiaz EA, Denis P, et al (1999) Beta-secretase cleavage of Alzheimer's amyloid precursor protein by the transmembrane aspartic protease BACE. Science 286: 735–741. 10531052 10.1126/science.286.5440.735

[48] Yan R, Vassar R (2014) Targeting the beta secretase BACE1 for Alzheimer's disease therapy. Lancet Neurol 13: 319–329. 10.1016/S1474-4422(13)70276-X 24556009 PMC4086426

[49] Tamagno E, Guglielmotto M, Monteleone D, Vercelli A, Tabaton M (2012) Transcriptional and post-transcriptional regulation of beta-secretase. IUBMB Life 64: 943–950. 10.1002/iub.1099 23180460

[50] Gong B, Pan Y, Vempati P, Zhao W, Knable L, Ho L, et al (2013) Nicotinamide riboside restores cognition through an upregulation of proliferator-activated receptor-gamma coactivator 1alpha regulated beta-secretase 1 degradation and mitochondrial gene expression in Alzheimer's mouse models. Neurobiol Aging 34: 1581–1588. 10.1016/j.neurobiolaging.2012.12.005 23312803 PMC3632303

[51] Singh AK, Pati U (2015) CHIP stabilizes amyloid precursor protein via proteasomal degradation and p53-mediated trans-repression of beta-secretase. Aging Cell 14: 595–604. 10.1111/acel.12335 25773675 PMC4531073

